# Sexual Dysfunction in Women with Inflammatory Bowel Disease

**DOI:** 10.3390/jcm14072236

**Published:** 2025-03-25

**Authors:** Daniel Mayrhofer, Jenny Shtokman-Shehab, Clemens Dejaco, Daniela Dörfler, Nadja Valenta-Taschler, Nora Rosenberg, Florian Heinzl, Johannes Ott, Klara Rosta

**Affiliations:** 1 Department of Obstetrics and Gynaecology, Clinical Division of Gynaecologic Endocrinology and Reproductive Medicine, Medical University of Vienna, 1090 Vienna, Austria; daniel.mayrhofer@meduniwien.ac.at (D.M.);; 2 Department of Internal Medicine III, Division of Gastroenterology and Hepatology, Medical University of Vienna, 1090 Vienna, Austria

**Keywords:** Crohn’s disease, ulcerative colitis, sexual health, female sexual functionality index

## Abstract

**Background:** Crohn’s disease (CD) and ulcerative colitis (UC) are chronic inflammatory bowel diseases (IBDs) characterized by various clinical symptoms including abdominal pain, diarrhea, fatigue, and extraintestinal manifestations, which negatively affect a patient’s quality of life. Both mainly occur in adolescence and young adulthood and therefore affect women in their sexually active period. The aim of this study was to assess the effect of IBD on female sexuality and attitudes towards contraception. **Methods:** A prospective cross-sectional survey study was conducted at the Medical University of Vienna, Austria. Data were collected using a self-designed questionnaire, which included questions on demographics, gynecological patient history, contraceptive choices, and fertility, as well as the Female Sexual Functionality Index (FSFI). **Results:** A total of 83 female patients with IBD (CD: n = 47, UC: n = 36) and 340 healthy control participants between the ages of 18 and 50 years were investigated. Demographic parameters did not differ between the groups; however, mean FSFI scores were significantly lower in the patient group (*p* < 0.001). Significantly fewer patients in the IBD group used contraception (*p* = 0.008). No significant differences regarding conception rates and infertility rates were noted between patients with IBD and control participants (*p* = 0.533 and *p* = 0.506, respectively). **Conclusions:** Female sexuality is significantly impaired in patients with IBD. Women with IBD do not receive sufficient information regarding contraception and should be screened for sexual dysfunction to optimize their quality of life.

## 1. Introduction

Inflammatory bowel disease (IBD), including both Crohn’s disease (CD) and ulcerative colitis (UC), is a chronic condition characterized by relapsing inflammation of the digestive system [1]. It develops as a result of an interaction between genetic and environmental factors that alter the immune response and cause multiple gastrointestinal symptoms, mainly prolonged diarrhea with abdominal pain and bloody stool [2,3]. With an incidence of 5–6 newly diagnosed patients per 100,000 people per year, approximately 50,000 patients suffer from IBD in Austria [4]. Chronic diseases can impact the quality of life of patients, including their sexuality, sexual health, and family planning.

Sexual dysfunction includes disorders that lead to persisting stress and challenges in daily life, and which require the care of a multidisciplinary team of specialists [5]. It is generally grouped into four categories: sexual desire disorders, sexual arousal disorders, dyspareunia, and difficulties achieving orgasm. Mental and physical health problems have been identified as major predictors of sexual dysfunction among reproductive age women, while age, education, sexual frequency, parity, and being in a relationship did not seem to have a clear protective or risk-reducing effect [6]. Female sexual dysfunction is common, with a prevalence of up to 43% [7]. Recent studies show that the prevalence of sexual dysfunction is even higher in women with IBD, ranging up to 50% [5,8]. Several biological and psychological factors contribute to increased rates of sexual dysfunction in patients with IBD. Active disease symptoms, such as abdominal pain or diarrhea, are linked to impaired sexual function [9]. Depression, anxiety disorders, and a negative body image and self-perception are regular issues patients with IBD face, which can reduce sexual satisfaction and function [10,11].

Based on a cohort study on 2569 patients with IBD, 71% of whom were women, the sexual interest of patients with IBD is almost identical to that of the general population; however, sexual satisfaction is significantly lower for both genders [12]. A cross-sectional questionnaire study [13] found that depression, anxiety, and sexual dysfunction were all significantly higher in the IBD group as compared to the control group. They also discovered that active disease had a detrimental effect on the mental and sexual well-being of patients. A recent systematic literature review found that more than half of female patients with IBD suffer from sexual dysfunction. Interestingly, the review showed that an inactive disease phase has little to no impact on fertility rates, but misconceptions and psychological issues lead to voluntary childlessness among patients with IBD [8].

There is still a lack of adequate counseling regarding fertility, pregnancy preparation, and pregnancy care for patients with IBD. Unfortunately, misconceptions regarding infertility and IBD can affect contraceptive choices and family planning [14]. Disease activity at the time of conception is an important predictor of disease course during pregnancy, as well as pregnancy outcomes and complications. Special care is required for patients receiving teratogenic medication, as well as for patients with high disease activity. The lack of counseling leads to the use of ineffective contraception methods or patients steering away from using contraception altogether [15]. Inadequate contraceptive choices may result in unplanned pregnancy without conscious preparation, which can lead to the application of potentially teratogenic drugs during gestation, flare-ups of the underlying disease, and elevated risk of fetal and maternal complications [16]. However, multiple studies have shown that oral contraceptive intake does not correlate with increased disease activity or risk of IBD relapse [16,17,18]. According to the Center for Disease Control and Prevention (CDC) and the World Health Organization (WHO), first-line recommendations for contraception in patients suffering from IBD include levonorgestrel-releasing intrauterine devices (IUDs), copper IUDs, and contraceptive implants [19].

Sexual dysfunction, family planning, and fertility, as well as the use of contraceptives, are closely interrelated topics. We aimed for a comprehensive evaluation of these topics in order to approximate the complexity of the real-world setting of patients with IBD, which has not been investigated so far in its entirety. Our results may contribute to a deeper understanding of the problems and needs of this group of patients. Therefore, the aim of this study was to evaluate the impact of IBD on the sexual function of female patients compared to healthy women. Our secondary aim was to gather information on contraceptive choices, family planning, and reproductive concepts.

## 2. Materials and Methods

### 2.1. Patients and Healthy Controls

This prospective, cross-sectional, single-center, questionnaire study was conducted between July 2020 and January 2021 at the Medical University of Vienna, Vienna, Austria, and was approved by the ethical committee of the Medical University of Vienna (IRB number 1209/2020). All women with a diagnosis of CD or UC and with no, mild, or well-managed comorbidities (e.g., asthma, arterial hypertension), aged between 18 and 50 years, were eligible for inclusion. The following patients were excluded: patients <18 or >50 years, patients with an unconfirmed diagnosis of IBD, patients with depression or an anxiety disorder, those with severe comorbidities (e.g., cancer, heart disease, diabetes, chronic obstructive pulmonary disease, rheumatic or other autoimmune diseases), and those with an inability to read or fill out the questionnaire on their own. The recruitment of the study group was performed in the outpatient clinic of the Department of Gastroenterology of the Medical University of Vienna. After applying the inclusion and exclusion criteria and obtaining written informed consent, each participant received a paper-based questionnaire to fill out, as well as an invitation to recruit healthy female peers. The control group was recruited through an online questionnaire distributed via snowball sampling. Each participant in the IBD group received a link to the questionnaire and was encouraged to share it with her healthy female peers, who could then further distribute it to their own healthy female peers.

### 2.2. Data Collection

The participants received the questionnaire, which was filled out anonymously by the control group and pseudo-anonymously by the study group. After agreeing to participate each index patient received a patient ID (a number between 1 and 88) and filled out the consent form, including their name and birthdate. The patient ID was written on the first page of the questionnaire, thus ensuring pseudo-anonymity. Patient history was partially evaluated from the questionnaire and from medical records in order to optimize data quality and minimize missing data. Gynecological data were mostly provided through the self-reported questionnaire, as those parameters are not routinely evaluated at the department of gastroenterology. Variables relating to disease activity were recorded in patient history electronically and double checked through three individuals (J.S-S., N.V., and N.R.) to ensure reliable data quality. Disease activity was defined by using the Harvey–Bradshaw Index for Crohn’s diseases and the Mayo Clinical Disease Activity Index for UC at the Department of Gastroenterology because these indices have direct relevance to real-time disease activity and therapy response. For the comparison of inflammation parameters in the study group, we used C-reactive protein (CRP) and calprotectin levels obtained during routine blood testing in the IBD outpatient clinic. CRP levels were categorized into three groups; CRP < 1 mg/dL representing no inflammation, 1–4 mg/dL representing mild inflammation, and CRP levels ≥ 4 mg/dL indicating severe active inflammation, as suggested by Vermeire et al. [20]. Miscarriage was defined as unintended loss of pregnancy, while abortion was classified as intended pregnancy termination. Information on pharmaceutical treatment regimens was provided by the patients and double-checked by investigating the patient files.

### 2.3. Questionnaire Design

First, a 69-item questionnaire was designed specifically for the patient group to query baseline characteristics including demographic information, medical history, and detailed gynecological history. All of the questionnaires are provided as supplement materials, both in English and German. Some of the questions, which were specific to IBD, were excluded in the questionnaire for the control group. Secondly, the 19-item Female Sexual Functionality Index (FSFI) questionnaire, originally created by Rosen et al. [21] and later validated in German by Berner et al. [22], was used to assess sexuality, satisfaction with sex life, and sexual function and dysfunction. The FSFI has been proven to be valid and reliable by Meston et al. [23]; clinical cutoff scores were developed by Wiegel et al. [24], suggesting that a score below 26 should be considered a risk for sexual dysfunction. General demographic characteristics such as age, height, weight, history of smoking and alcohol use, and relationship status were recorded for all participants. Among patients, data were collected regarding the course of their disease, including type and date of diagnosis, disease complications, disease activity, and present and previous treatment. For assessing the disease activity for CD, the Harvey–Bradshaw Index was used [25]. For UC, the Mayo Clinical Disease Activity Index was applied [26]. Both indices are widely used by gastroenterologists to categorize disease severity and were filled out by all outpatients of the gastroenterological department as part of routine care [26,27].

### 2.4. Statistical Analysis

Statistical analyses were performed with the SPSS software package, version 27.0 (IBM Corp: Armonk, NY, USA). Nominal variables were expressed as percentages (%), while numeric variables were reported as mean ± standard deviation. For comparing normally distributed variables with equal variance between the three groups (UC, CD, control), we used standard ANOVA with Tukey post hoc test. In cases of non-homogeneity, we employed Welch’s ANOVA and chose the Games–Howell test for post hoc testing. When normally distributed continuous variables were compared between the two groups, UC and CD, the independent-samples *t*-test was used. Normality of the distributions was tested using the Shapiro–Wilk test, while Levene’s test was used to check for homogeneity of variances across the three groups. The Kruskal–Wallis test was used to compare non-normally distributed numerical variables among all three groups, while the Mann–Whitney U test was used to compare numerical variables with non-normal distribution between two groups. The chi-square test was utilized when two nominal variables were compared. A *p* value of <0.05 was considered statistically significant.

## 3. Results

### 3.1. General Characteristics

A total of 423 participants were included. Five patients from the initial IBD group (n = 88) were excluded due to missing data or unsecured diagnosis of IBD. IBD was diagnosed by specialists at the IBD outpatient clinic of the Department of Gastroenterology, Medical University of Vienna. Finally, out of 83 patients with IBD, 47 women (56.6%) suffered from CD and 36 (43.4%) suffered from UC. Seven individuals from the initial control group (n = 347) were excluded for not meeting the inclusion/exclusion criteria. Thus, the final control group consisted of 340 voluntary participants without IBD.

The mean age in the CD group was 35.7 ± 9.5 years, 33.4 ± 7.5 years in the UC group, and 32.8 ± 9.2 years for the control group. No significant difference regarding the mean age between the IBD groups and the control group were observed. Significant differences were found in alcohol consumption and relationship status. No statistically significant differences were observed between the groups regarding other baseline characteristics, e.g., body mass index (BMI), and smoking history. Baseline characteristics are shown in Table 1.

### 3.2. Disease Characteristics

Disease characteristics are shown in Supplementary Table S1. Considering the yearly hospitalization rate, flare-ups, and yearly outpatient visits, no significant differences between patients with CD vs. UC were observed. When comparing disease activity, the number of patients with CD in remission was significantly higher (*p* = 0.002) with 53% compared to 14% in the UC group. Therefore, 47% of patients with CD presented with active disease compared to 86% of patients with UC. When comparing the inflammation parameter calprotectin, no significant difference was observed with both groups showing 54% of patients with calprotectin levels ≥ 250 µg/mg. Seventy-two percent of patients with CD had no inflammation (<1 mg/dL) compared with 97% of patients in the UC group (*p* = 0.012). Calprotectin and CRP levels were associated with disease activity in both groups, as all of the patients with severe disease had calprotectin levels > 250 µg/mg, in contrast to around 50% in the lower activity group. None of the patients in remission had CRP levels > 4mg/dL, compared to 3% in the mild disease group, 8% in the moderate disease group, and 25% in the severe disease group. In total, 89% of patients with CD and 92% of patients with UC required pharmaceutical treatment.

### 3.3. Sexuality and Sexual Functionality

The control group showed a significantly higher FSFI score compared to the CD and UC groups (29.5 ± 5.0 in the control vs. 12.6 ± 5.1 in the CD and 11.4 ± 5.0 in the UC groups, respectively; *p* < 0.001). No differences between the IBD groups were observed (*p* = 0.478). A comparison based on disease severity among patients with IBD showed no significant difference (*p* = 0.853). The number of sexual partners was significantly lower among the CD group compared with the other two groups (*p* < 0.001). The majority of patients with CD (49%) had ≤3 sexual partners in their lifetime, whereas most patients in the UC and control group had 4–10 sexual partners (43% and 37%, respectively). Across all three groups, the majority of individuals were between 16 and 20 years of age at the first time of sexual intercourse. The frequency of monthly intercourse was significantly different between all three groups, with higher rates in the control group (*p* < 0.001). Although no differences were observed between the CD and UC group (*p* = 0.940), the difference between each disease group and the control participants was significant (*p* = 0.001). Table 2 shows the findings regarding sexuality and sexual function. The monthly intercourse frequency was also compared among patients with IBD in different stages of disease severity (see Table 3).

### 3.4. Fertility and Contraception

No significant differences were found among all three groups concerning the mean age of menarche, rate of unplanned pregnancies, and time passed since the last visit to the gynecologist. Out of all participants who were trying to conceive, 60% in the CD group, 38% in the UC group, and 62% in the control group had been trying for over 12 months, although these findings were not statistically significant (*p* = 0.506). Among all the women who had already been pregnant, there was no statistical difference between the groups concerning the ability to conceive without intervention (*p* = 0.533) and number of abortions (*p* = 0.41). No significant difference was found comparing each of the groups separately to each other. Noteworthy, the patient group showed a significantly higher number of miscarriages (*p* = 0.025). Data regarding fertility are summarized in Table 4.

A significantly higher number of patients in the IBD group did not use any contraception (CD: 45%; UC: 44% vs. control: 27%; *p* < 0.008). Interestingly, contraceptive methods that were significantly more common in the IBD group included the contraceptive patch (*p* = 0.002), contraceptive injection (*p* < 0.001), contraceptive implant (*p* = 0.004), and spermicides (*p* = 0.001). The only method significantly more common in the control group was the copper IUD (*p* = 0.047). Emergency contraception use at least twice in a lifetime was significantly more common among control participants as compared to patients with IBD (30% vs. 6% for CD and 14% for UC). Table 5 shows the distribution of contraceptive methods in all three groups.

## 4. Discussion

The results of our study confirm that IBDs have a strong impact on the sexual function of affected patients. Sexual dysfunction was defined as an FSFI score below 26 as suggested by Wiegel et al. [24]. Of note, all of the patients with IBD in this study suffered from sexual dysfunction, their FSFI scores were significantly lower in all aspects of sexual function, when compared to the control group. In contrast, 30% of the control group scored below 26 points. The frequency of monthly intercourse was also considerably lower in the IBD group. Bel et al. [9] conducted a study on the sexual dysfunction of 168 female and 119 male patients with IBD and found no significant differences regarding the prevalence of sexual dysfunction between patients and control subjects. However, the authors found that patients with active disease scored lower than patients in remission or the control group. In our study, FSFI scores did not differ between patients with different disease severity stages. This may reflect the predominantly mild disease activity or remission among our outpatient cohort, with only a small subset experiencing moderate or severe disease. The limited sample size of index individuals could also contribute to these findings. In addition, Bel et al. also included male patients, who generally suffer from less sexual dysfunction than females [28]. Several chronic conditions, such as migraine, multiple sclerosis, and autoimmune rheumatic diseases, significantly impact sexual function, with high rates of sexual dysfunction reported among affected women (93.75%, 72.3%, and 63%, respectively) [29,30,31]. Our study suggests that IBD similarly affects sexual function, with a substantial proportion of female IBD patients experiencing sexual dysfunction. Consistent with our findings, Marin et al. reported that half of the female patients experienced decreased desire and satisfaction following an IBD diagnosis, alongside a higher prevalence of sexual dysfunction [32]. However, it is important to consider that FSFI scores can be influenced by multiple factors beyond medical conditions, including psychological well-being, socioeconomic status, education, lifestyle, and sociocultural background [33,34].

When comparing the monthly sexual intercourse frequency, the IBD group had lower frequencies than the control group, although there was no significant difference in the percentage of women who were in committed relationships longer than 12 months. Moody et al. [35] compared the frequency of sexual intercourse of 150 patients with IBD and 122 controls and found no significant differences. A possible explanation might be that Moody et al. also included male patients, and that the mean age and number of participants in a committed relation in their study was higher than in our population.

Multiple questions regarding contraception and fertility were included to assess the impact IBD has on the patient’s contraceptive choices and fertility. Significantly more women with IBD do not use any contraception at all compared to the control group. Contraceptive choices in patients with IBD are often influenced by misconceptions regarding the effect of contraception on disease activity or fertility. Unfortunately, contraceptive counseling is often neglected due to the inadequate knowledge of healthcare professionals concerning the specificities of patients with IBD [16]. Uncertain and contradictory information regarding safety, effectiveness, and possible side effects of contraceptives methods might lead to patients opting out of using any contraception at all.

Despite being one of the lowest ranked methods (failure rate of 18% over 1 year of use [36]), the most frequently used contraceptive in the IBD group was the condom. A safe and effective contraceptive method is especially important for patients with IBD with high disease activity, as they often take teratogenic medications and have a higher risk of miscarriage, fetal growth restriction, and preterm delivery by unplanned pregnancies [16]. The most effective contraceptive methods for patients with IBD according to the CDC and WHO are hormonal IUDs, copper IUDs, and contraceptive implants [19]. In our study, the low percentage of patients using IUDs indicates that women with IBD generally lack sufficient information about contraceptives. The CDC guideline for contraceptive use in IBD was released in 2016 and not updated since then [37]. It is important to consider that patients with IBD, besides the conventional risks that limit contraceptive choices, such as the Pearl Index or risk of venous thromboembolism, have multiple factors which influence the choice of contraception including disease activity, level of malabsorption, remaining bowel length after surgery, and the influence of progesterone on gut motility. Recent studies suggest that low-dose combined oral contraceptives (COCs) have been proven to be safe and effective in cases of low disease activity [38]. Effective oral uptake and absorption might be reduced by malabsorption, ileal resections, or after extensive bowel operations. Nonetheless, a meta analysis associated COC usage with the increased risk of developing IBD [39]. However, the total duration, dose of COC exposure, and the risk for development of IBD need to be better characterized. The individual risk of venous thromboembolism should always be assessed when prescribing COC given the history of the patient and recent activity of the disease. Individualized choice of contraception or using better defined subgroups when suggesting contraception might improve the sexuality and quality of life of these patients. Toomey et al. [40] found that more than 30% of patients with IBD had never discussed family planning and contraception with their doctors. Gawron et al. [16] found that contraceptive counseling was initiated more than half of the time by patients themselves, and despite some patients receiving methotrexate, there was only limited documentation on the contraceptive choices of patients. This confirms our hypothesis that there is a paucity of contraceptive information for female patients with IBD. According to our findings, most participants received reproductive counseling from their gynecologist (UC 78%, CD 87%, controls 85%); only 6% of patients with UC and 4% of patients with CD received information about contraception from their internal medicine specialist. The data on fertility show a difference among the three groups regarding the number of miscarriages, with the control group having significantly fewer miscarriages than the IBD groups. There were no differences in the number of abortions. Our findings show that about the same percentage of patients with IBD have unplanned pregnancies as the control group. According to a systematic review [41], patients with well-managed IBD do not have reduced fertility rates compared to the general population. A recent meta-analysis, including 28 studies by Kim et al. [42], shows an OR of 2.27 for spontaneous abortion and stillbirth in patients with IBD with active disease compared to patients with inactive IBD. These results indicate that disease activity is a main factor for adverse pregnancy outcomes.

The strength of our study lies in its comprehensive approach to sexuality with the inclusion of attitudes of contraceptive usage, as well as the detailed history about the underlying inflammatory disease and its therapy. Our finding, in concert with other studies, underlines the interconnected nature of sexuality, fertility, and reproductive potential, emphasizing the importance of careful counseling, pregnancy planning, and contraception for women with IBD.

Several limitations must be acknowledged. The small sample size, particularly in the IBD group, precludes definitive conclusions about subgroup differences, as well as about the impact of disease severity and immunosuppressive medications on sexuality. Additionally, only outpatients with relatively mild disease severity were included; results might have reached statistical significance if hospitalized patients had been considered, particularly regarding disease activity. In our study, real-time disease activity assessment was performed by using the Harvey–Bradshaw Index for Crohn’s disease and the Mayo Clinical Disease Activity Index for UC. The Montreal classification is not recorded at the outpatient clinic as it does not facilitate immediate clinical decision making but rather helps for the estimation of long-term prognosis. The lack of the data about the Montreal classification limits insights into disease-specific effects, such as localization on sexual health.

The control group was recruited via snowball sampling, a non-probability method that may introduce biases related to lifestyle, education, and social similarity. Lastly, the study focused on women aged 18–50, excluding the potential effects of (post-)menopause on sexual function in IBD.

Due to the relative paucity of evidence regarding sexual function in post-menopausal women [43], this could be an interesting direction for future research with patients with IBD.

## 5. Conclusions

This cross-sectional study confirms the negative impact of inflammatory bowel disease on the sexual and reproductive health of female patients. Women with IBD should be sufficiently educated regarding contraception and fertility. Interdisciplinary counseling should provide appropriate information about sexuality and contraceptive methods to this special group of patients.

## Figures and Tables

**Table 1:** Baseline characteristics of the study groups and the control group: CD and UC groups and control group.

	CD Group (n = 47)	UC Group (n = 36)	Control Group (n = 340)	*p*-Value
Mean age	35.7 ± 9.5	33.4 ± 7.5	32.8 ± 9.2	0.118
BMI	23.3 ± 5.6	23.7 ± 4.2	23.3 ± 4.8	0.087
Smoking history (%)	15	22	20	0.651
Alcohol consumption (%)	6	11	33	<0.001
In a relationship <1 year (%)	6	11	16	0.186
In a relationship >1 year (%)	62	50	60	

Abbreviation: BMI: body mass index; CD: Crohn’s disease; UC: ulcerative colitis.

**Table 2:** Comparison of sexuality and sexual function between the CD and UC groups and the control group.

	CD Group (n = 47)	UC Group (n = 36)	Control Group (n = 340)	*p*-Value
Mean FSFI Score	12.6 ± 5.1	11.4 ± 5.0	29.5 ± 5.0	<0.001
Number of sexual partners				0.030
<4 sexual partners (%)	49	43	26	
4–10 sexual partners (%)	45	43	37	
>10 sexual partners (%)	6	14	37	
Age at first sexual intercourse				<0.001
<16 years (%)	6	25	33	
16–20 years (%)	77	61	42	
>20 years (%)	17	14	26	
Monthly intercourse frequency				<0.001
0 times/month (%)	43	42	18	
1–5 times/month (%)	45	44	39	
6–10 times/month (%)	2	5	25	
>10 times/month (%)	11	8	18	

Abbreviation: CD: Crohn’s disease; CRP: C-reactive protein; FSFI: Female Sexual Functionality Index; UC: ulcerative colitis.

**Table 3:** Female Sexual Functionality Index score and intercourse frequency in relation to disease severity.

	Remission (n = 30)	Mild Disease (n = 35)	Moderate Disease (n = 13)	Severe Disease (n = 4)
Mean FSFI Score	12.0 ± 5.4	11.8 ± 4.7	13.6 ± 5.2	12.3 ± 5.8
Monthly intercourse frequency				
0 times/month (%)	33	40	62	50
1–5 times/month (%)	50	49	23	50
6–10 times/month (%)	3	6	0	0
>10 times/month (%)	14	5	15	0

Abbreviation: FSFI: Female Sexual Functionality Index.

**Table 4:** Aspects of fertility and family planning in patients with CD and UC and the healthy control group.

	CD Group (n = 47)	UC Group (n = 36)	Control (n = 340)	*p*-Value
≥1 unplanned pregnancies (%)	13	11	8	0.513
Mean age of menarche	13.3 ± 1.8	12.9 ± 1.3	13.0 ± 1.4	0.228
Last visit to the gyn. > 1 year ago (%)	17	19	26	0.763
Conception without intervention possible (%)	96 *	100 *	94 *	0.533
≥1 miscarriages (%)	44 *	47 *	24 *	0.025
≥1 abortions (%)	13 *	16 *	24 *	0.410
Unfulfilled desire to conceive ≥ 1 year (%)	60 **	38 **	62 **	0.506

* Out of the women, who had been pregnant before; ** out of the women, who are trying to conceive. Abbreviation: CD: Crohn’s disease; UC: ulcerative colitis.

**Table 5:** Contraceptive choices in patients with CD and UC and healthy controls.

	CD (n = 47)	UC (n = 36)	Control (n = 340)	*p*-Value
Contraception method				
No contraception (%)	45	44	27	0.008
Contraceptive patch (%)	12 *	0 *	1 *	0.002
Natural family planning (%)	4 *	10 *	5 *	0.577
Contraceptive injection (%)	8 *	10 *	<1 *	<0.001
Contraceptive implant (%)	4 *	5 *	0 *	0.004
Coitus interruptus (%)	12 *	5 *	5 *	0.312
Vaginal ring (%)	0 *	5 *	1 *	0.315
Hormonal IUD (%)	8 *	0 *	10 *	0.321
Combined pill (%)	23 *	15 *	22 *	0.755
Condom (%)	46 *	55 *	40 *	0.381
Copper IUD (%)	8 *	5 *	22 *	0.047
Mini pill (%)	0 *	5 *	3 *	0.559
Spermicide (%)	0 *	5 *	<1 *	0.001
≥2 times use of emergency contraception (%)	6	14	30	0.003

* Out of the women, who are currently using contraception. Abbreviation: CD: Crohn’s disease; IUD: intrauterine device; UC: ulcerative colitis.

## Data Availability

The data are available from the corresponding author upon reasonable request.

## Supplementary Materials

The following supporting information can be downloaded at https://www.mdpi.com/article/10.3390/jcm14072236/s1. Table S1: Disease activity and treatment of the CD and UC groups.
