# Supplementary material for: Sexual Dysfunction in Women with Inflammatory Bowel Disease
Source: J Clin Med. 2025 Mar 25;14(7):2236. doi: 10.3390/jcm14072236 (PMC11989570; doi:10.3390/jcm14072236)
Supplement: Supplementary file 1 [file jcm-14-02236-s001.zip › jcm-3467038-supplementary.pdf]

Supplementary Materials

Table S1. Disease activity and treatment of CD and UC group.

	CD Group (n = 47)	UC Group (n = 36)	*p*-Value
Mean age at diagnosis	23.0 ± 8.3	23.9 ± 7.3	0.610
Zero hospitalizations in last 1 year (%)	87	94	0.156
Flare-up in the past 6 months (%)	57	61	0.737
Mean hospital visits in last 1 year	4.0 ± 2.0	4.3 ± 2.3	0.561
Disease activity			
Remission (%)	53	14	
Mild disease (%)	30	60	0.002
Moderate disease (%)	15	17	
Severe disease (%)	2	9	
Calprotectin >250 μg/mg (%)	54	54	0.996
CRP < 1 mg/dL (%)	72	97	
CRP 1–4 mg/dL (%)	21	3	0.012
CRP > 4 mg/dL (%)	6	0	
Type of medication			
No medication (%)	11	8	
5-ASA (%)	6	14	
Biological (%)	43	25	
5-ASA + biological (%)	19	19	0.188
Biological + immunosuppressant (%)	4	3	
Biological + corticosteroid (%)	2	3	
Other combinations (%)	15	28	
Other organ system involvement			
No involvement (%)	26	56	0.005
Skin involvement (%)	36	25	0.277
Liver diseases (%)	9	6	0.606
Other parts of gastrointestinal tract (%)	45	22	0.033
Eye infection (%)	2	3	0.372
Joint pain (%)	2	3	0.372

Abbreviation: 5 ASA 5-aminosalicylic acid; CD: Crohn's disease; CRP: C-reactive protein; UC: ulcerative colitis.
